# New Biochemical Pathway May Control Erection

**DOI:** 10.1100/tsw.2001.29

**Published:** 2001-05-01

**Authors:** Thomas M. Mills

**Affiliations:** Department of Physiology, Medical College of Georgia, Augusta 30912-3000, USA

**Keywords:** penis, penile erection, Rho-kinase, vascular smooth muscle, erectile dysfunction (impotence)

## Abstract

Thirty million men in the U.S. suffer from erectile dysfunction (ED) defined by their inability to achieve or maintain a penile erection sufficient for intercourse. An unestimated number of women also suffer from sexual dysfunction resulting from many of the same causes that lead to ED in men. There are a variety of treatments available for ED including intracavernosal injection, transurethral therapy, surgery, vacuum therapy, and oral medication. Unfortunately, not all patients benefit from these currently available forms of therapy, and side effects are not uncommon. Sildenafil (Viagra) has been a highly successful drug for the treatment of ED but it does not work in all men [5]. Some may experience a variety of side effects, and Viagra is contraindicated to some cardiac medications. These problems point to the need for new and different approaches to the treatment of sexual problems.

A recent publication may signal a previously untapped avenue for the treatment of ED. This study, published in *Nature Medicine*, reported on the activity of the RhoA/Rho-kinase, calcium-sensitizing pathway in the penis and the finding that specific inhibition of Rho-kinase activity causes erection [2]. This suggests that the activity of the RhoA/Rho-kinase pathway mediates vasoconstriction in the cavernosal circulation so that its inhibition reduces the vasoconstriction and permits smooth muscle relaxation and erection. Normally, a combination of contraction of the smooth muscle in the arterioles carrying blood to the erectile tissue and contraction of the smooth muscle in the erectile tissue keeps blood flow low and maintains the penis in the non-erect state [1,6]. The vasoconstriction may be partly mediated by RhoA, a monomeric small GTP binding protein that is activated when it binds GTP. Activated RhoA, in turn, activates Rho-kinase, and this enzyme causes increased phosphorylation, and thus inhibition, of myosin light chain (MCL) phosphatase [4]. With a suppression of the activity of MLC phosphatase, the level of phosphorylated MLC remains high so that myosin can interact with alpha actin, resulting in smooth muscle contraction. It follows that inhibition of Rho-kinase leads to increased MLC phosphatase activity, dephosphorylation of MLC phosphate, and smooth muscle relaxation. As the smooth muscle relaxes, blood flow increases and erection results. In the published studies, Rho-kinase activity was inhibited with the experimental drug Y-27632, and the treatment resulted in erection [2]. This drug was developed by the Welfide Corporation, Osaka Japan for use in the treatment of hypertension. Studies in experimentally induced, hypertensive rats showed that Y-27632, when given systemically or orally, lowered blood pressure in these rats. There was little effect of Y-27632 when given to normotensive rats [8].

As reported in *Nature Medicine* [2], the erectile response was measured as the blood pressure within the corpora cavernosa of the penis (intracavernosal pressure, ICP) relative to the simultaneously measured mean arterial pressure (MAP). ICP increased within 2 or 3 min of injection of Y-27632 into the cavernous sinuses of the penis. The drug response was dose dependent: a dose of 2 nmol/kg of the drug gave a minimal increase in ICP; the increase was maximal at 200 nmol/kg and did not increase further when the dose was raised to 400 nmol/kg. Notably, in the dose range of 2–200 nmol/kg, MAP was not significantly lowered. Also of importance was the finding that the response to the inhibitor occurred even when nitric oxide (NO)-mediated pathways had been blocked with specific inhibitors of nitric oxide synthase (L-NAME, L-NNA) or inhibitors of guanylate cyclase (methylene blue, ODQ). These experiments provide strong evidence that the response to the Rho-kinase inhibitor is independent of the NO-cGMP mediated pathway of vasorelaxation which is considered to be the principle mechanism by which erection occurs. The finding that inhibition of Rho-kinase with Y-27632 leads to erection in the absence of NO suggests that NO-mediated vasorelaxation is only part of the story. Inhibition of the Rho-kinase–mediated vasoconstrictor pathways may also be a significant part of erection. Other aspects of the published report include the demonstration of Rho-kinase protein expression in smooth muscle extracted from the penis and a confirmation of the relaxing effects of Y-27632 in isolated strips of rat penile tissue incubated in a muscle bath.

Despite the demonstration of significant RhoA/Rho-kinase activity in the penis, several aspects and questions remain to be answered. For example, the biochemical control of this pathway is not known in the penis; do potent vasoconstrictors in the cavernosal circulation, such as norepinephrine [7] and endothelin-1 [3] regulate activity of the RhoA/Rho-kinase pathway? Is the activity constitutive and therefore independent of these agents? How does erection occurs in the presence of the very high levels of vasoconstrictor activity in the cavernosal circulation? Does NO-induced vasorelaxation override Rho-kinase activity, or is another mechanism involved? Despite these questions, the presence of Rho-kinase activity and the finding that its inhibition leads to erection may have great clinical relevance in the treatment of ED. The challenge in the further development of this finding lies in the determination of the optimal route of delivery of the inhibitors, development of strategies for targeting Rho-kinase activity in the penis without affecting its activity in other vascular beds, and the evaluation of side effects. Regardless of these difficulties, the potential for a new and innovative approach to treatment of ED based on the inhibition of vasoconstriction is exciting.

| | | |
|---|---|---|
| Received: | February | 15, 2001 |
| Accepted: | March | 26, 2001 |
| Published: | May | 1, 2001 |
